# Prevalence of and factors associated with stroke in hypertensive patients in Thailand from 2014 to 2018: A nationwide cross-sectional study

**DOI:** 10.1038/s41598-021-96878-4

**Published:** 2021-09-02

**Authors:** Wittawat Chantkran, Janeyuth Chaisakul, Ram Rangsin, Mathirut Mungthin, Boonsub Sakboonyarat

**Affiliations:** 1 Department of Pathology, Phramongkutklao College of Medicine, Level 6, Her Royal Highness Princess Bejaratana Building, 317 Ratchawithi Road, Ratchathewi District, Bangkok, 10400 Thailand; 2 Department of Pharmacology, Phramongkutklao College of Medicine, Bangkok, 10400 Thailand; 3 Department of Military and Community Medicine, Phramongkutklao College of Medicine, Level 5, Her Royal Highness Princess Bejaratana Building, 317 Ratchawithi Road, Ratchathewi District, Bangkok, 10400 Thailand; 4 Phramongkutklao College of Medicine, Bangkok, 10400 Thailand

**Keywords:** Health care, Neurology, Risk factors

## Abstract

Stroke is a significant cause of death and disability. In Thailand, it imposes a major health burden, and the prevalence of stroke is increasing, particularly in patients with hypertension (HT), diabetes mellitus (DM), and dyslipidemia (DLP). We aimed to determine the trends in the prevalence of stroke and the associated factors among Thai patients with HT. Nationwide cross-sectional studies were conducted annually in 2014, 2015 and 2018 based on data obtained from the Thailand DM/HT study. Nationally, representative patients with HT in Thailand were sampled with stratified one-stage cluster sampling. A total of 104,028 participants were included in this study. The prevalence of stroke remained constant, with prevalence rates of 4.0%, 3.8%, and 3.9% in 2014, 2015 and 2018, respectively (*p* for trend = 0.221). Our findings suggested that the management of stroke patients who are covered by the universal coverage scheme should be evaluated. Effective interventions, including promoting smoking cessation, attenuating cholesterol levels, and controlling blood pressure should be provided to hypertensive patients to prevent ischemic stroke. Young adults with HT should be more concerned about the possibility of stroke. The use of prophylactic low-dose aspirin should be carefully monitored to prevent hemorrhagic stroke.

## Introduction

Worldwide, stroke is one of the leading causes of death and long-term disability^1^. Based on data from the Global Burden of Diseases, Injuries, and Risk Factors Study (GBD) 2017^2^, the age-standardized incidence of patients with stroke declined by 11% (from 0.17 to 0.15%) worldwide from 1990 to 2017^3^. This decline may have resulted from more aggressive control of the risk factors through the adoption of preventive measures. Similarly, the global age-standardized stroke mortality rate decreased by 33.4% (from 0.12 to 0.08%), and the disability-adjusted life years lost decreased by 31% (from 2.39 to 1.66%) from 1990 to 2017. In contrast, the global age-standardized prevalence of stroke has increased by 3.1% (from 1.26 to 1.30%). However, from 1990 to 2017, the prevalence increased only in upper-middle-income countries, with an average growth rate of 11% (from 1.40 to 1.55%), whereas it decreased by 3% (from 1.03 to 1.00%) and 8% (from 1.26 to 1.16%) in low- and high-income countries, respectively, and remained unchanged (1.08%) in lower-middle-income countries.

Thailand is an upper-middle-income country located in Southeast Asia. Over the past few decades, Thailand has gradually transformed from an agricultural to an industrialized nation. The economic and health transitions in addition to urbanization have modified many aspects of the lifestyles of the Thai people, including the introduction of unhealthy diets and reductions in the level of physical activity^4,5^. As a consequence, the prevalence of metabolic syndrome and cardiovascular diseases, including stroke, has increased. Stroke has become a major health burden and the leading cause of death in Thailand, with a higher prevalence in males^5^. The mean age of patients at the onset of ischemic stroke is approximately 65 years^6^. From 1994 to 1996, the prevalence of stroke in Thailand was 1.12%, with the highest prevalence found in the central region^7^. A study conducted over the period from 2004 to 2006 demonstrated that the prevalence of stroke was approximately 1.88% among Thai individuals aged 45 years and older^5,8^. In 2014, it was reported that the prevalence of stroke in Thailand was 1.3%^9^.

Many risk factors for stroke are modifiable. Among these, hypertension (HT) is an important risk factor for stroke^10–13^. Other risk factors include diabetes mellitus (DM), dyslipidemia (DLP), and overweight^10,14^. Lifestyle modifications, including smoking cessation, physical activity participation, healthy diet consumption and body weight control, can reduce mortality from stroke^15^. However, information on the distribution of the prevalence of stroke according to geographic region, hospital level, or health coverage scheme in Thailand is limited. In this study, we aimed to determine the prevalence of stroke in Thai patients with HT visiting the Ministry of Public Health and Bangkok Metropolitan Administration Hospitals from 2014 to 2018 based on information from the Thailand Diabetes Mellitus/Hypertension (DM/HT) database and identify the associated factors. The outcomes of this study potentially support the development of preventive strategies and promote the capacity of healthcare professionals to counsel patients with stroke in Thailand.

## Methods

### Study designs and subjects

Cross-sectional surveys were conducted once per year in 2014, 2015 and 2018. After receiving permission from the National Health Security Office (NHSO) and Medical Research Network of the Consortium of Thai Medical School (MedResNet), the database “An Assessment on Quality of Care among Patients Diagnosed with Type 2 Diabetes and Hypertension Visiting the Ministry of Public Health and Bangkok Metropolitan Administration Hospitals in Thailand” (Thailand DM/HT) was accessed, and the relevant data were retrieved^16^. Patients visiting subdistrict (health-promoting), district (community), provincial (general) and regional hospitals nationwide were included in the Thailand DM/HT study, with the aim of encompassing all hospital levels under the Ministry of Public Health. Among the 1,098 hospitals, a total of 28 regional hospitals, 80 general hospitals, 883 community hospitals and 107 health-promoting hospitals participated. In this study, the inclusion criteria were HT, age of 18 years or older, and medical treatment in the abovementioned hospitals during the previous 12 months. Patients who had participated in a clinical trial were excluded. Nationally and provincially, representatives of patients with HT in Thailand were sampled proportional to the population size with a stratified one-stage cluster sampling method.

### Data collection

At each clinic, health care personnel invited patients with a pre-existing diagnosis of HT to participate in the study. The patients were asked to sign a consent form to allow the investigators to review and abstract their medical records. A standardized case report form (CRF) was used for medical record abstractions. The CRF was completed by a well-trained registered nurse using a standard protocol and was sent to the MedResNet central data management unit in Nonthaburi, Thailand. To transfer the CRF to the electronic dataset, an automated scan-to-database software solution was used to extract data and convert them to database records^16^. The collected data comprised demographics, weight, height, body mass index (BMI), smoking status, systolic blood pressure (SBP), diastolic blood pressure (DBP), stroke history, stroke subtypes, stroke comorbidities, including DLP, DM, gout, renal insufficiency, atrial fibrillation (AF), and history of low-dose aspirin use. In this study, the database from the Thailand DM/HT study was used for analysis. Based on secondary data, stroke was determined according to the International Classification of Diseases and Related Health Problems, Tenth Revision (ICD-10) codes^17^ I60-I64 for overall stroke, I63 for ischemic stroke, and I60-I62 for hemorrhagic stroke recorded by clinicians. Similarly, stroke comorbidities were determined according to the ICD-10 codes^17^ E78 for DLP, E11 for DM, M10 for gout, N18-N19 for renal insufficiency, and I48 for AF. Current smokers were defined as those who had smoked within the previous 12 months. Nonsmokers were patients who had never smoked or had smoked fewer than 100 cigarettes^18^ in their lifetime. Ex-smokers were defined as those who had smoked at least 100 cigarettes in their lifetime but had quit smoking for at least 12 months. BMI was calculated as the body weight in kilograms divided by the height in meters squared [weight (kg)/height (m)^2^].

### Statistical analysis

The data were analyzed using Stata Statistical Software: Release 17 (StataCorp LLC., College Station, TX, USA). Demographic characteristics were analyzed with descriptive statistics. The outcomes are presented as numbers and percentages for categorical data and as the means and standard deviations (SDs) for continuous data. The prevalence of stroke was assessed using descriptive statistics and is reported as the percentage with the associated 95% confidence interval (95% CI). The *p* for trend was calculated using the chi-square test for trend. The chi-square test and Student’s *t*-test were used to compare categorical and continuous variables, respectively. Univariate and multivariate logistic regression analyses were performed to determine the associated factors for stroke. Sex, age, geographic region, hospital level, healthcare coverage scheme, smoking, DM, DLP, renal insufficiency, AF, BMI, and uncontrolled HT were included in the multivariate logistic regression analyses of the factors associated with ischemic stroke. Sex, age, geographic region, hospital level, healthcare coverage scheme, smoking, DLP, renal insufficiency, BMI, and the use of prophylactic low-dose aspirin were adjusted in the final model to determine the factors associated with hemorrhagic stroke. The magnitudes of the associations obtained from univariate and multivariate analyses are represented as crude odds ratios (ORs) and adjusted odds ratios (AORs), respectively, with the corresponding 95% CIs. The level of statistical significance was set at *p* < 0.05.

### Ethical considerations

The Thailand DM/HT study was approved by the Thai National Health Security Office Institutional Review Board. This study was reviewed and approved by the Institutional Review Board, Royal Thai Army Medical Department (approval number S086h/63). This study was conducted in accordance with the principles of the Declaration of Helsinki. Informed consent was obtained from all individual participants included in the study.

## Results

### Demographic characteristics

A total of 104,028 Thai patients with HT were included in the study, with 33,227 (31.9%) in 2014, 32,440 (31.2%) in 2015 and 38,361 (36.9%) in 2018. Females accounted for 62.1% of all participants. The average ages of the participants were 63.8 ± 11.7, 63.9 ± 11.8 and 64.6 ± 11.8 years, and the average durations of HT since diagnosis were 6.4 ± 4.0, 6.7 ± 4.2 and 6.8 ± 4.5 years in 2014, 2015, and 2018, respectively. The majority of participants were patients visiting community hospitals (72.6%). Most of the patients were covered under the universal coverage scheme (74.2%). The demographic characteristics of the study participants by year are presented in Table 1.

**Table 1:** Demographic characteristics of participants (n = 104,028).

Year	2014	2015	2018
Characteristics	n = 33,227	n = 32,440	n = 38,361
	n (%)	n (%)	n (%)
**Sex**			
Male	12,400 (37.3)	12,432 (38.3)	14,593 (38.0)
Female	20,814 (62.7)	20,007 (61.7)	23,768 (62.0)
**Age (years)**			
18–30	51 (0.2)	52 (0.2)	69 (0.2)
30–39	524 (1.6)	553 (1.7)	558 (1.5)
40–49	3259 (9.8)	3124 (9.6)	3281 (8.6)
50–59	8259 (24.9)	8017 (24.7)	9006 (23.5)
60–69	10,065 (30.3)	9899 (30.5)	12,194 (31.8)
70–79	7886 (23.8)	7625 (23.5)	8968 (23.4)
≥ 80	3120 (9.4)	3168 (9.8)	4285 (11.2)
Mean ± SD	63.8 ± 11.7	63.9 ± 11.8	64.6 ± 11.8
**Geographic region**			
Northeast	7387 (22.2)	7477 (23.0)	10,562 (27.5)
North	9834 (29.6)	8198 (25.3)	8854 (23.1)
Central	11,351 (34.2)	11,753 (36.2)	12,422 (32.4)
South	4655 (14.0)	5012 (15.5)	6523 (17.0)
**Hospital level**			
Regional hospital	2352 (7.1)	2744 (8.5)	2488 (6.5)
General hospital	5868 (17.7)	6592 (20.3)	6614 (17.2)
Community Hospital	25,007 (75.3)	23,104 (71.2)	27,375 (71.4)
Health Promoting Hospital	n/a	n/a	1884 (4.9)
**Occupation**			
Agriculturist	11,540 (34.7)	11,109 (34.2)	13,145 (34.3)
Retirement	11,501 (34.6)	10,937 (33.7)	13,968 (36.4)
Employee	5533 (16.7)	5420 (16.7)	5779 (15.1)
Private business	1486 (4.5)	1732 (5.3)	1838 (4.8)
Government officer	1535 (4.6)	1811 (5.6)	2016 (5.3)
Others	1632 (4.9)	1431 (4.4)	1615 (4.2)
**Religion**			
Buddhist	29,966 (95.8)	29,722 (95.6)	35,441 (94.8)
Islamic	1230 (3.9)	1315 (4.2)	1851 (5.0)
Christian	51 (0.2)	38 (0.1)	81 (0.2)
Others	43 (0.1)	7 (0.0)	4 (0.0)
**Scheme**			
Universal coverage	24,892 (75.0)	23,513 (72.5)	28,781 (75)
Government officers	6415 (19.3)	6766 (20.9)	7323 (19.1)
Social Security Insurance	1578 (4.8)	1695 (5.2)	1861 (4.9)
Others	293 (0.9)	466 (1.4)	396 (1.0)
Diabetes mellitus	4351 (13.1)	4277 (13.2)	5743 (15.0)
Dyslipidemia	20,627 (62.1)	20,544 (63.3)	23,741 (61.9)
Gout	2598 (7.8)	2485 (7.7)	3012 (7.9)
Renal insufficiency	4037 (12.2)	3800 (11.7)	12,781 (12.3)
**Body mass index (kg/m2)**			
Mean ± SD	24.9 ± 4.6	25.0 ± 4.7	25.0 ± 4.7
**Duration of hypertension (years)**			
Mean ± SD	6.4 ± 4.0	6.7 ± 4.2	6.8 ± 4.5
**Systolic blood pressure (mmHg)**			
Mean ± SD	132.0 ± 15.1	133.2 ± 15.2	133.9 ± 14.4
**Diastolic blood pressure (mmHg)**			
Mean ± SD	75.6 ± 10.6	76.3 ± 10.7	75.9 ± 10.5

n/a = not available.

### Trends in the prevalence of stroke among Thai patients with hypertension

Table 2 illustrates the trends in the prevalence of stroke by sex, age group, and geographic region. From 2014 to 2018, the overall prevalence of stroke among Thai patients with HT remained unchanged. The prevalences were 4.0%, 3.8%, and 3.9% for overall stroke (*p* for trend = 0.221); 3.6%, 3.4%, and 3.5% for ischemic stroke (*p* for trend = 0.517); and 0.5%, 0.4% and 0.4% for hemorrhagic stroke (*p* for trend = 0.033) in 2014, 2015, and 2018, respectively. Overall, although the prevalence of stroke remained constant over the 5 years in both males (*p* for trend = 0.15) and females (*p* for trend = 0.686), the prevalence was higher in males. From 2014 to 2018, the prevalence of overall stroke was approximately 5.6% to 6.0% in males, while it was approximately 2.7% to 2.8% in females. Strikingly, a significant increasing trend in the prevalence of overall stroke was found in patients under the age of 40 years, with prevalence rates of 0.9%, 1.3%, and 2.6% (*p* for trend = 0.02) in 2014, 2015, and 2018, respectively. In contrast, in patients aged between 50 and 59 years, the prevalence of overall stroke significantly decreased from 3.5% in 2014 to 3.4% in 2015 and further decreased to 2.8% in 2018 (*p* for trend = 0.007). This pattern was also seen in ischemic stroke. With regard to the geographic distribution of the prevalence of stroke, the prevalence of overall stroke significantly increased from 3.0% in 2014 to 3.6% in 2018 (*p* for trend < 0.005) in patients living in the northeastern part of Thailand. In contrast, among patients residing in the central part of Thailand, the prevalence of overall stroke significantly declined from 4.4% in 2014 to 3.7% in 2018 (*p* for trend = 0.005). Again, it was observed that this pattern only existed in ischemic stroke. The prevalence of hemorrhagic stroke significantly decreased from 0.7% in 2014 to 0.4% in 2018 (*p* for trend = 0.001) among patients living in the northern part of Thailand.

**Table 2:** The trends in the prevalence of overall, ischemic, and hemorrhagic strokes among Thai patients with hypertension over the period from 2014 to 2018.

Characteristics	Overall stroke			*p* for trend	Ischemic stroke			*p* for trend	Hemorrhagic stroke			*p* for trend
	2014	2015	2018		2014	2015	2018		2014	2015	2018	
	% (95% CI)	% (95% CI)	% (95% CI)		% (95% CI)	% (95% CI)	% (95% CI)		% (95% CI)	% (95% CI)	% (95% CI)	
**Sex**												
Male	6.0 (5.6–6.4)	5.6 (5.2–6.0)	5.6 (5.2–6.0)	0.150	5.3 (4.9–5.7)	5.0 (4.6–5.4)	5.0 (4.6–5.4)	0.308	0.8 (0.7–1.0)	0.6 (0.5–0.8)	0.7 (0.5–0.8)	0.116
Female	2.8 (2.6–3.1)	2.7 (2.4–2.9)	2.8 (2.6–3.0)	0.686	2.6 (2.4–2.8)	2.4 (2.2–2.7)	2.6 (2.4–2.8)	< 0.001	0.3 (0.3–0.4)	0.2 (0.2–0.3)	0.3 (0.2–0.3)	0.144
**Age (years)**												
< 40	0.9 (0.1–1.6)	1.3 (0.4–2.2)	2.6 (1.3–3.8)	0.020	0.5 (0.0–1.0)	1.0 (0.2–1.8)	2.0 (0.8–3.0)	0.024	0.3 (0.0–0.8)	0.3 (0.0–0.8)	0.8 (0.1–1.0)	0.261
40–49	2.3 (1.8–2.8)	2.2 (1.7–2.7)	2.5 (2.0–3.0)	0.595	1.8 (1.4–2.3)	1.9 (1.4–2.4)	2.0 (1.5–2.5)	0.552	0.5 (0.3–0.8)	0.3 (0.1–0.4)	0.6 (0.3–0.8)	0.727
50–59	3.5 (3.1–3.9)	3.4 (3.0–3.8)	2.8 (2.4–3.1)	0.007	3.1 (2.7–3.5)	2.9 (2.6–3.3)	2.4 (2.1–2.7)	0.009	0.5 (0.3–0.6)	0.5 (0.3–0.6)	0.4 (0.2–0.5)	0.237
60–69	4.1 (3.7–4.5)	3.9 (3.5–4.3)	3.7 (3.4–4.1)	0.134	3.6 (3.3–4.0)	3.4 (3.1–3.8)	3.4 (3.1–3.7)	0.362	0.6 (0.4–0.7)	0.5 (0.3–0.6)	0.4 (0.3–0.6)	0.093
≥ 70	5.1 (4.6–5.5)	4.6 (4.2–5.0)	5.1 (4.7–5.5)	0.766	4.6 (4.2–5.0)	4.3 (4.0–4.8)	4.8 (4.4–5.1)	0.566	0.5 (0.4–0.6)	0.3 (0.2–0.4)	0.4 (0.3–0.5)	0.134
**Geographic region**												
Northeast	3.0 (2.6–3.4)	2.5 (2.2–2.9)	3.6 (3.3–4.0)	0.005	2.8 (2.4–3.2)	2.3 (1.9–2.6)	3.3 (3.0–3.7)	0.013	0.2 (0.1–0.4)	0.3 (0.1–0.4)	0.3 (0.2–0.4)	0.510
North	4.3 (3.9–4.7)	4.0 (3.6–4.5)	3.8 (3.4–4.2)	0.087	3.7 (3.3–4.1)	3.6 (3.2–4.1)	3.5 (3.1–3.9)	0.419	0.7 (0.5–0.9)	0.4 (0.3–0.6)	0.4 (0.3–0.5)	0.001
Central	4.4 (4.0–4.7)	4.4 (4.0–4.8)	3.7 (3.3–4.0)	0.005	3.8 (3.5–4.2)	3.9 (3.5–4.2)	3.3 (3.0–3.7)	0.029	0.5 (0.4–0.7)	0.5 (0.4–0.7)	0.4 (0.3–0.5)	0.218
South	4.4 (3.8–5.0)	3.9 (3.4–4.4)	4.7 (4.2–5.2)	0.340	3.9 (3.4–4.5)	3.7 (3.2–4.2)	4.0 (3.6–4.5)	0.650	0.6 (0.4–0.8)	0.2 (1.0–3.7)	0.7 (0.5–0.9)	0.321
Total	4.0 (3.8–4.3)	3.8 ( 3.6–4.0)	3.9 ( 3.7–4.1)	0.221	3.6 (3.4–3.8)	3.4 (3.2–3.6)	3.5 (3.3–3.7)	0.517	0.5 (0.4–0.6)	0.4 (0.3–0.5)	0.4 (0.4–0.5)	0.033

The *p* for trend is calculated using the chi-square test for trend and the level of statistical significance is set at *p* for trend < 0.05.

### Associated factors of stroke among Thai patients with hypertension

Univariate and multivariate regression analyses were performed to identify the independent factors for ischemic stroke and hemorrhagic stroke among Thai patients with HT from 2014 to 2018. After adjusting for potential confounders, factors associated with ischemic stroke were male sex, older age, geographic region, hospital level, healthcare coverage schemes, smoking, comorbid DLP, AF, low BMI, and uncontrolled HT (Table 3). For hemorrhagic stroke, the independent associated factors were male sex, younger age, geographic region, hospital level, healthcare coverage schemes, low BMI, and the use of prophylactic low-dose aspirin (Table 4).

**Table 3:** Univariable and multivariable analyses for factors associated with ischemic stroke among Thai patients with hypertension.

Factors	Total	Ischemic stroke	Crude odds ratio	95%CI	*p*-value	Adjusted odds ratio	95%CI	*p*-value
	n	n (%)						
**Year**								
2014	33,227	1192 (3.6)	1.00					
2015	32,440	1112 (3.4)	0.95	0.88–1.04	0.267	–	–	–
2018	38,361	1340 (3.5)	0.97	0.90–1.05	0.496	–	–	–
**Sex**								
Female	64,589	1634 (2.5)	1.00			1.00		
Male	39,425	2009 (5.1)	2.07	1.94–2.21	< 0.001	1.88	1.73–2.04	< 0.001
**Age (years)**								
< 40	1807	21 (1.2)	1.00					
40–49	9664	185 (1.9)	1.66	1.05–2.61	0.029			
50–59	25,282	710 (2.8)	2.46	1.59–3.80	< 0.001			
60–69	32,158	1115 (3.5)	3.06	1.98–4.72	< 0.001			
≥ 70	35,117	1613 (4.6)	4.09	2.66–6.31	< 0.001			
Mean ± SD	64.1 ± 11.8	67.3 ± 11.1	1.03	1.02–1.03	< 0.001	1.01	1.01–1.02	< 0.001
**Geographic region**								
Northeast	25,426	728 (2.9)	1.00			1.00		
North	26,886	971 (3.6)	1.27	1.15–1.40	< 0.001	1.09	0.99–1.21	0.094
Central	35,526	1314 (3.7)	1.3	1.19–1.43	< 0.001	1.16	1.05–1.28	0.004
South	16,190	631 (3.9)	1.38	1.23–1.53	< 0.001	1.19	1.06–1.34	0.004
**Hospital level**								
Community Hospital	75,486	2266 (3)	1.00			1.00		
General hospital	19,074	905 (4.7)	1.61	1.49–1.74	< 0.001	1.59	1.46–1.74	< 0.001
Regional hospital	7584	460 (6.1)	2.09	1.88–2.31	< 0.001	2.00	1.78–2.26	< 0.001
Health Promoting Hospital	1884	13 (0.7)	0.23	0.13–0.39	< 0.001	0.27	0.15–0.46	< 0.001
**Healthcare coverage scheme**								
Universal health coverage	77,186	2768 (3.6)	1.00			1.00		
Government officer	20,504	716 (3.5)	0.97	0.90–1.06	0.518	0.75	0.68–0.82	< 0.001
Social Security Insurance	5134	126 (2.5)	0.68	0.57–0.81	< 0.001	0.69	0.56–0.84	< 0.001
Others	1155	33 (2.9)	0.79	0.56–1.12	0.186	0.73	0.50–1.08	0.111
**Smoking**								
Never	81,948	2513 (3.1)	1.00			1.00		
Current	4449	217 (4.9)	1.62	1.41–1.87	< 0.001	1.25	1.08–1.46	0.004
Ex–smoker	12,433	724 (5.8)	1.96	1.80–2.13	< 0.001	1.32	1.19–1.46	< 0.001
**Diabetes mellitus**								
No	89,657	3106 (3.5)	1.00			1.00		
Yes	14,371	538 (3.7)	1.08	0.99–1.19	0.091	1.02	0.91–1.13	0.778
**Dyslipidemia**								
No	39,116	1016 (2.6)	1.00			1.00		
Yes	64,912	2628 (4.0)	1.58	1.47–1.70	< 0.001	1.68	1.55–1.83	< 0.001
**Gout**								
No	95,933	3333 (3.5)	1.00					
Yes	8095	311 (3.8)	1.11	0.99–1.25	0.084	–	–	–
**Renal insufficiency**								
No	91,247	3050 (3.3)	1.00			1.00		
Yes	12,781	594 (4.6)	1.41	1.29–1.54	< 0.001	1.08	0.98–1.20	0.117
**Atrial fibrillation**								
No	102,894	3522 (3.4)	1.00			1.00		
Yes	1134	122 (10.8)	3.40	2.81–4.12	< 0.001	2.68	2.17–3.3	< 0.001
**Body mass index (kg/m**^**2**^**)**								
18.5–22.9	28,260	1174 (4.2)	1.00					
< 18.5	6517	304 (4.7)	1.13	0.99–1.28	0.066			
23–24.99	19,510	673 (3.4)	0.82	0.75–0.91	< 0.001			
25–29.99	33,088	897 (2.7)	0.64	0.59–0.70	< 0.001			
≥ 30	13,181	283 (2.1)	0.51	0.44–0.58	< 0.001			
Mean ± SD	25.0 ± 4.7	23.9 ± 4.4	0.94	0.94–0.95	< 0.001	0.95	0.95–0.96	< 0.001
**Uncontrolled hypertension**								
SBP < 140 mmHg and DBP < 90 mmHg	68,696	2304 (3.4)	1.00			1.00		
SBP ≥ 140 mmHg or DBP ≥ 90 mmHg	35,332	1340 (3.8)	1.14	1.06–1.22	< 0.001	1.14	1.06–1.22	0.001

The level of statistical significance is set at *p* < 0.05.

**Table 4:** Univariable and multivariable analyses for factors associated with hemorrhagic stroke among Thai patients with hypertension.

Factors	Total	Hemorrhagic stroke	Crude odds ratio	95%CI	*p*–value	Adjusted odds ratio	95%CI	*p-*value
	n	n (%)						
**Year**								
2014	33,227	175 (0.5)	1.00					
2015	32,440	127 (0.4)	1.26	1.02–1.57	0.033	–	–	–
2018	38,361	160 (0.4)	0.94	0.74–1.19	0.593	–	–	–
**Sex**								
Female	64,589	178 (0.3)	1.00			1.00		
Male	39,425	283 (0.7)	2.62	2.17–3.16	< 0.001	2.61	2.07–3.30	< 0.001
**Age (years)**								
< 40	1807	9 (0.5)	1.00					
40–49	9664	44 (0.5)	0.91	0.45–1.88	0.806			
50–59	25,282	112 (0.4)	0.89	0.45–1.76	0.735			
60–69	32,158	160 (0.5)	1.00	0.51–1.96	0.998			
≥ 70	35,117	137 (0.4)	0.78	0.40–1.54	0.477			
Mean ± SD	64.1 ± 11.8	63.5 ± 11.2	1.00	0.99–1.00	0.217	0.98	0.97–0.99	< 0.001
**Geographic region**								
Northeast	25,426	68 (0.3)	1.00			1.00		
North	26,886	137 (0.5)	1.91	1.43–2.56	< 0.001	1.74	1.28–2.36	< 0.001
Central	35,526	174 (0.5)	1.84	1.39–2.43	< 0.001	1.54	1.13–2.09	0.006
South	16,190	83 (0.5)	1.92	1.39–2.65	< 0.001	1.82	1.28–2.57	0.001
**Hospital level**								
Community Hospital	75,486	239 (0.3)	1.00			1.00		
General hospital	19,074	162 (0.8)	2.70	2.21–3.29	< 0.001	2.65	2.11–3.33	< 0.001
Regional hospital	7584	57 (0.8)	2.38	1.78–3.19	< 0.001	2.16	1.51–3.08	< 0.001
Health Promoting Hospital	1884	4 (0.2)	0.67	0.25–1.809	0.427	0.8	0.30–2.17	0.667
**Healthcare coverage scheme**								
Universal health coverage	77,186	357 (0.5)	1.00			1.00		
Government officer	20,504	75 (0.4)	0.79	0.62–1.01	0.064	0.56	0.42–0.75	< 0.001
Social Security Insurance	5134	22 (0.4)	0.93	0.60–1.43	0.728	0.61	0.38–0.99	0.043
Others	1155	7 (0.6)	1.31	0.62–2.78	0.478	0.9	0.37–2.18	0.81
**Smoking**								
Never	81,948	303 (0.4)	1.00			1.00		
Current	4449	31 (0.7)	1.89	1.31–2.74	0.001	1.12	0.76–1.66	0.556
Ex–smoker	12,433	94 (0.8)	2.05	1.63–2.59	< 0.001	1.18	0.90–1.55	0.220
**Diabetes mellitus**								
No	89,657	403 (0.4)	1.00					
Yes	14,371	59 (0.4)	0.91	0.69–1.20	0.515	–	–	–
**Dyslipidemia**								
No	39,116	154 (0.4)	1.00			1.00		
Yes	64,912	308 (0.5)	1.21	0.99–1.46	0.058	1.14	0.92–1.42	0.235
**Gout**								
No	95,933	421 (0.4)	1.00					
Yes	8095	41 (0.5)	1.16	0.84–1.59	0.380	–	–	–
**Renal insufficiency**								
No	91,247	389 (0.4)	1.00			1.00		
Yes	12,781	73 (0.6)	1.34	1.04–1.72	0.022	1.23	0.93–1.63	0.14
**Atrial fibrillation**								
No	102,894	455 (0.4)	1.00					
Yes	1134	7 (0.6)	1.40	0.66–2.96	0.380	–	–	–
**Body mass index (kg/m**^**2**^**)**								
18.5–22.9	28,260	136 (0.5)	1.00					
< 18.5	6517	32 (0.5)	1.02	0.69–1.5	0.918			
23–24.99	19,510	89 (0.5)	0.95	0.73–1.24	0.694			
25–29.99	33,088	110 (0.3)	0.69	0.54–0.89	0.004			
≥ 30	13,181	36 (0.3)	0.57	0.39–0.82	0.002			
Mean ± SD	25.0 ± 4.7	24.1 ± 4.5	0.96	0.94–0.98	< 0.001	0.95	0.92–0.97	< 0.001
**Uncontrolled hypertension**								
SBP < 140 mmHg and DBP < 90 mmHg	68,696	308 (0.4)	1.00					
SBP ≥ 140 mmHg or DBP ≥ 90 mmHg	35,332	154 (0.4)	0.97	0.8–1.18	0.774	–	–	–
**Low**–**dose aspirin use**								
No	74,241	271 (0.4)	1.00			1.00		
Yes	29,787	191 (0.6)	1.76	1.46–2.12	< 0.001	1.78	1.45–2.20	< 0.001

The level of statistical significance is set at *p* < 0.05.

## Discussion

To the best of our knowledge, this is the first and largest epidemiological study in Southeast Asia to focus on stroke and its associated factors among Thai patients with HT. These results revealed the constant trends in the prevalence of stroke among Thai patients with HT from 2014 to 2018. The prevalence of overall stroke in this population ranged from 3.8 to 4.0% (Table 2). Unsurprisingly, the prevalence of ischemic stroke was higher than that of hemorrhagic stroke. In patients with HT, the prevalence of stroke was 5.1% in Madrid, Spain^19^, and 11.6% in Southern Piauí, Brazil^20^. In Thailand, the prevalence of overall stroke in patients with HT was relatively low. This may be the result of improvements in the stroke management system. Indeed, in Thailand, a stroke fast track system was initiated and has been improved over the past two decades^21^. As a consequence, more acute stroke patients are treated within the golden period.

Previously, the central part of Thailand was reported to have the highest prevalence of stroke, followed by the southern, northern, and northeastern regions; the northeastern region has been reported to have the lowest prevalence of stroke in Thailand^8^. In this study, however, the geographic distribution of the prevalence of stroke in patients with HT was slightly different. We found that ischemic stroke was most prevalent in the southern part of Thailand, whereas the northeastern region still had the lowest stroke prevalence (Table 2). However, there was a significant increasing trend in the prevalence of ischemic stroke in patients with HT residing in the northeastern part of Thailand from 2014 to 2018. With regard to hemorrhagic stroke, the prevalence was also lowest in the northeastern region (Table 2), and the risk of stroke nearly doubled in patients with HT residing in other regions of the country (Table 4). The most plausible explanation of the lowest prevalence of both ischemic and hemorrhagic strokes being found in the northeastern part of Thailand is that agriculture is the main occupation of people residing in this area with a lower rate of industrialization. Their lifestyles are still conservative; for example, healthy diets consisting of mainly rice and vegetables and regular physical activity related to their occupation may lower the risk of stroke. However, further investigation is required to identify the associated factors contributing to a significant increasing trend in the prevalence of ischemic stroke in this area and the change from the central to the southern region where the highest prevalence of ischemic stroke was observed.

Interestingly, in Thai patients with HT, we found that the prevalence of both ischemic (Table 3) and hemorrhagic strokes (Table 4) was higher in patients who were admitted to regional and provincial hospitals than in those admitted to community hospitals. This can be explained by the fact that stroke patients are likely to be referred to tertiary care centers. Specialists and essential medical facilities are not available in health-promoting and community hospitals; as a result, patients with stroke in such hospitals may have limited access to neurologists. At present, Thailand has fewer than 400 active neurologists. More than two-thirds of this specialized group lives in Bangkok and the surrounding provinces^5^. The concentration of neurologists in Bangkok results in a shortage of neurologists across the country, particularly in rural areas. In addition, we found that the highest prevalence of both ischemic (Table 3) and hemorrhagic strokes (Table 4) was observed in hypertensive patients who were under the universal health coverage scheme, which is the major healthcare coverage scheme in Thailand. The most plausible explanation is that there are imbalances between the demand and supply of treatment for stroke that affect the management system within the universal health coverage scheme. The need for longitudinal studies on the outcomes of stroke at different hospital levels and the impact of universal health coverage schemes on the outcome of stroke is suggested. Collectively, these findings indicated that stroke should be a focus in regional and provincial hospitals and that opportunities to prevent stroke should be considered by providing more intensive preventive strategies to hypertensive patients, especially those who are under the universal health coverage scheme.

The present study revealed that male sex was a risk factor for both ischemic (Table 3) and hemorrhagic strokes (Table 4) in Thai patients with HT. These results were consistent with previous studies performed in southwestern China^10^ and in Thailand, particularly in patients older than 35 years old^22^. An analysis of 471,971 participants from the UK Biobank cohort study demonstrated that the incidence of stroke was higher among males^23^. However, this remains controversial, as opposite results have been reported. In the USA, the incidence and prevalence of stroke were higher in females^24,25^. It was reported that estrogen was a protective factor against the development of ischemic stroke^26^. Therefore, the higher prevalence of stroke in males may be related to a lower level of female hormones. However, menopausal and postmenopausal females may lose this protective effect, which results in an increased susceptibility to ischemic brain changes^26^.

Although stroke can occur at any age, it is mainly a disease of the elderly population. We found that the risk of ischemic stroke increased with increasing age group among Thai patients with HT in a dose–response relationship (Table 3). The average age of patients at the onset of ischemic stroke in Thailand, according to the Thai Stroke Registry, is approximately 65 years^6^. This age is consistent with the average age of onset of ischemic stroke reported in other developing countries^27–29^. Nevertheless, from 2014 to 2018, the prevalence of overall and ischemic strokes continuously increased in patients with HT who were younger than 40 years (Table 2). In addition, the present study also found an inverse dose–response relationship between age and the risk of hemorrhagic stroke (Table 4). These results indicated that young Thai people with HT should be more concerned about the possibility of stroke. Interestingly, in Western countries, the proportion of patients with hemorrhagic stroke is dramatically higher in the young adult subpopulation: 15–20% of the general stroke population has hemorrhagic stroke, while 40–55% of young adult diagnosed with stroke has hemorrhagic stroke^30–33^. In a large American study, fivefold and 2.33-fold increased risks of hemorrhagic stroke were observed in young adults who abused amphetamine and cocaine, respectively^34^. Mechanistically, amphetamine can induce cerebral vasculitis^35,36^, whereas cocaine is involved in hypertensive surges following its administration^37,38^. However, it was revealed that, rather than the direct effects of the drugs, the higher risk of hemorrhagic stroke in those young adults was potentially associated with underlying vascular malformations^39^. The proportion of patients with hemorrhagic stroke has not been well investigated among young Asians.

The present study demonstrated inverse dose–response relationships between BMI and the risk of both ischemic (Table 3) and hemorrhagic strokes (Table 4) in Thai patients with HT, with a higher risk of stroke in hypertensive patients who had lower BMI values. These results were consistent with those of a previous study conducted in 67,086 American patients with DM^40^. More clinical and molecular insights are, however, still needed to explain these findings.

Heavy metals and other toxins in tobacco smoke promote vascular endothelial dysfunction and inflammation, ultimately resulting in atherosclerosis^41^. In addition, smoking also promotes a global procoagulant state^42^ which results in a decrease in cerebral blood flow^43^, leading to blood clot formation and ischemic stroke development. While the relationship between intracerebral hemorrhage and smoking is weak and inconsistent, an increased risk of subarachnoid hemorrhage tends to be associated with the increased incidence of aneurysms seen in smokers^41^. A strong association between smoking and stroke risk has been reported, with current smokers having at least a two- to fourfold higher risk of stroke than nonsmokers or ex-smokers who quit smoking more than 10 years prior^41^. A number of studies have indicated that ex-smokers have a lower risk of stroke than current smokers^43–46^ and may have the same risk as nonsmokers^47,48^. Nonetheless, we found that both current smokers and ex-smokers with HT had an elevated risk of ischemic (Table 3) but not hemorrhagic (Table 4) stroke. Therefore, for the greatest benefit, smoking cessation should be listed in preventive rather than therapeutic strategies for stroke. Physiologically, smoking cessation can lead to a reversal of the procoagulant state to baseline. However, the full return to the risk status of a nonsmoker depends on various factors, including the level of atherosclerosis developed during smoking, the duration of smoking, and the duration since smoking cessation^41^. Unfortunately, this information was not available in our database.

HT is the most common and well-established risk factor for both ischemic and hemorrhagic strokes. In ischemic stroke, HT places a strain on the blood vessels and predisposes them to damage, which ultimately causes atherosclerosis^49^. HT is implicated in hemorrhagic stroke when a weakened blood vessel in the brain bursts and blood leaks into the brain. In our study, uncontrolled HT was identified as a factor associated with ischemic stroke in Thai patients with HT (Table 3). Therefore, controlling blood pressure is a critical management strategy that can reduce the risk of ischemic stroke in hypertensive patients. Unexpectedly, we found that uncontrolled HT was no longer associated with hemorrhagic stroke (Table 4). This result was supported by a previous study reporting that there was no association between the use of antihypertensive drugs and the outcome of cerebral small vessel disease^50^. However, these findings were only described in minority reports. The pooling of data from one hundred forty-seven randomized clinical trials demonstrated that 10 mmHg systolic and 5 mmHg diastolic reduction of blood pressure was associated with an average 41% reduction in stroke in all trials^51^. In fact, in a systemic review, all eleven case–control studies showed a positive correlation between HT and intracerebral hemorrhage, with an overall OR of 3.68 (95% CI, 2.52 to 5.38)^52^. The incidence of HT increases with age; hence, HT is a more common risk factor for intracerebral hemorrhage in older people. The stronger association between younger age groups of hypertensive patients and hemorrhagic stroke in this study potentially explains the lack of a correlation between HT and hemorrhagic stroke.

DLP is known to be a risk factor for atherosclerosis-related ischemic stroke. Our study revealed an association between comorbid DLP and the risk of ischemic stroke in Thai patients with HT (Table 3), indicating that lipid profiles should be regularly tested. However, a relationship between DLP and hemorrhagic stroke was not observed (Table 4). It has been reported that attenuating cholesterol levels can reduce the risk of stroke. Regarding statins versus placebo and more versus less statin therapy, the pooled results of twenty-six clinical trials demonstrated that there was a reduction in ischemic stroke with an overall relative risk (RR) of 0.79 (95% CI, 0.74 to 0.85), whereas there was a nonsignificant increase in hemorrhagic stroke with an overall RR of 1.12 (95% CI, 0.93 to 1.35) per 1 mmol/L reduction in low-density lipoprotein cholesterol (LDL-C)^53^. Gemfibrozil reduces serum triglycerides and raises high-density lipoprotein cholesterol (HDL-C). In the Veterans Affairs High-Density Lipoprotein Cholesterol Intervention Trial (VA-HIT) conducted in 2,531 men with coronary heart disease, it was reported that gemfibrozil reduced strokes by 31% (95% CI, 2% to 52%), and there were five and six hemorrhagic strokes in the placebo and gemfibrozil arms, respectively^54^.

In AF, the chaotic rhythm may cause blood to pool in the upper chambers of the heart and form clots that can dislodge and block blood flow to other organs, including the brain. The present study identified that AF was a strong associated factor for ischemic stroke in Thai patients with HT (Table 3). This result was consistent with previous studies^55–58^. However, it remains unclear whether occult AF is related to stroke. According to the Prevalence of Sub-Clinical Atrial Fibrillation Using an Implantable Cardiac Monitor in Patient With Cardiovascular Risk Factors (ASSERT-II) study, the rate of occurrence of subclinical AF in those with or without a history of stroke, systemic embolism, or transient ischemic attack was not significantly different^59^. With regard to stroke prevention, ongoing clinical trials are evaluating appropriateness and the risks and benefits of screening for occult AF and the use of anticoagulants in occult AF patients, including the Systematic ECG Screening for Atrial Fibrillation Among 75 Year Old Subjects in the Region of Stockholm and Halland, Sweden (STROKESTOP) trial (NCT01593553) and the Apixaban for the Reduction of Thromboembolism in Patients With Device-Detected Sub-Clinical Atrial Fibrillation (ARTESiA) trial (NCT01938248).

Given its cost-effectiveness and widespread availability, low-dose aspirin is a key therapeutic option for the secondary prevention of myocardial infarction and ischemic stroke^60^. Aspirin at a dose of 81 mg was the main prophylactic antiplatelet medication prescribed to the patients in our study. We found that low-dose aspirin use was a risk factor for hemorrhagic stroke in patients with HT (Table 4). Therefore, patients taking prophylactic low-dose aspirin should be closely monitored. In a systemic review, the pooled results of eight randomized clinical trials showed an association between low-dose aspirin use and an elevated risk of intracranial bleeding, with an overall RR of 1.37 (95% CI, 1.13 to 1.66)^61^. The pooling of data from four randomized clinical trials demonstrated that the strongest relationship was between low-dose aspirin use and subdural or extradural hemorrhage, with an overall RR of 1.53 (95% CI, 1.08 to 2.18). Moreover, it was concluded that Asians were at higher risk than other ethnicities.

There were some limitations of our study. First, patients with HT visiting university hospitals were not included; therefore, it is possible that the prevalence of stroke in hypertensive patients was underestimated. Second, the data were obtained from the Thailand DM/HT study. Therefore, the diagnosis of stroke and comorbidities in this study was based on the ICD-10 codes determined by clinicians. Third, the subclassifications of ischemic and hemorrhagic strokes were not further investigated because of the limited information available from the Thailand DM/HT database. Fourth, the study employed serial cross-sectional surveys; therefore, cause-and-effect relationships could not be identified between the associated risk factors and stroke. Although there were some data missing from the surveys, this was compensated for by the recruitment of a large number of participants from all geographic regions across the country. Hence, the associations between the outcomes and the risk factors were still robust. The strength of our study was that it was a large, nationwide epidemiological study focusing on stroke in Thai patients with HT. Our results can be generalized to the entire country and similar populations.

## Conclusion

We identified a constant trend in the prevalence of stroke among Thai patients with HT over the period from 2014 to 2018. Our findings suggested that the management of stroke patients who are covered by the universal health coverage scheme needs to be evaluated. Effective interventions, including promoting smoking cessation, attenuating cholesterol levels, and controlling blood pressure should be provided to all patients with HT to prevent ischemic stroke. Young adults with HT should be more concerned about the risk of stroke. The use of prophylactic low-dose aspirin should be more focused and carefully monitored to prevent hemorrhagic stroke.

## Data Availability

Data cannot be shared publicly because the data set contains identifying information, additionally, the data belong to the Thailand DM/HT study of the Medical Research Network of the Consortium of Thai Medical Schools (MedResNet); thus, there are ethical restrictions on the data set. Data are available from the Thai National Health Security Office (NHSO), Bangkok, Thailand (contact via sirikorn.k@nhso.go.th) for researchers who meet the criteria for access to confidential data. After permission, the researchers will be able to access the data set and variables at http://www.damus.in.th.

## Abbreviations

HT: Hypertension
DM: Diabetes mellitus
DLP: Dyslipidemia
BMI: Body mass index
AF: Atrial fibrillation
OR: Crude odds ratio
AOR: Adjusted odds ratio
RR: Relative risk
95% CI: 95% Confidence interval
